# Finite element model focused on stress distribution in the levator ani muscle during vaginal delivery

**DOI:** 10.1007/s00192-016-3126-1

**Published:** 2016-08-25

**Authors:** Ladislav Krofta, Linda Havelková, Iva Urbánková, Michal Krčmář, Luděk Hynčík, Jaroslav Feyereisl

**Affiliations:** 1 Institute for the Care of Mother and Child, Podolské nábřeží 157, 147 00 Praha, Czech Republic; 2 New Technologies—Research Centre, University of West Bohemia, Univerzitní 8, 306 14 Plzeň, Czech Republic

**Keywords:** MRI modeling, Levator ani muscle, Vaginal delivery

## Abstract

**Introduction and hypothesis:**

During vaginal delivery, the levator ani muscle (LAM) undergoes severe deformation. This stress can lead to stretch-related LAM injuries. The objective of this study was to develop a sophisticated MRI-based model to simulate changes in the LAM during vaginal delivery.

**Methods:**

A 3D finite element model of the female pelvic floor and fetal head was developed. The model geometry was based on MRI data from a nulliparous woman and 1-day-old neonate. Material parameters were estimated using uniaxial test data from the literature and by least-square minimization method. The boundary conditions reflected all anatomical constraints and supports. A simulation of vaginal delivery with regard to the cardinal movements of labor was then performed.

**Results:**

The mean stress values in the iliococcygeus portion of the LAM during fetal head extension were 4.91–7.93 MPa. The highest stress values were induced in the pubovisceral and puborectal LAM portions (mean 27.46 MPa) at the outset of fetal head extension. The last LAM subdivision engaged in the changes in stress was the posteromedial section of the puborectal muscle. The mean stress values were 16.89 MPa at the end of fetal head extension. The LAM was elongated by nearly 2.5 times from its initial resting position.

**Conclusions:**

The cardinal movements of labor significantly affect the subsequent heterogeneous stress distribution in the LAM. The absolute stress values were highest in portions of the muscle that arise from the pubic bone. These areas are at the highest risk for muscle injuries with long-term complications.

## Introduction

During vaginal delivery, the levator ani muscle (LAM) undergoes severe deformation. This stress can lead to stretch-related injuries, such as muscle tearing and striated muscle atrophy, owing to pudendal denervation [1], localized primarily to the region of the pubovisceral muscle (PVm) [2]. The PVm, also known as the pubococcygeus muscle, needs to be stretched during vaginal birth to over three times its original length [3]. This elongation is more than twice what the striated muscle can withstand without damage in a nonpregnant animal model [4].

On magnetic resonance imaging (MRI) the details of muscles are vivid, identifying the origin of this damage during vaginal birth [5]. Such muscle trauma, which is not visible after delivery by cesarean section, usually causes lifelong complications [6] and is associated with a significantly worse genital body image, poorer sexual health [7], and necessitates surgical treatment later in life [8].

It is important to understand the anatomy and physiology of the LAM during vaginal delivery to prevent or manage vaginal birth-related pelvic floor traumas. Several computer models of vaginal birth have been developed [4, 9], most of which are focused on the second stage of labor, starting from full dilatation of the cervix until birth of the fetus [10]. In this study cardinal movements of labor are often neglected.

Model geometry is commonly obtained from MRI [11]. The fetal head is sometimes oversimplified and replaced by an incompressible sphere [12, 13]. Bones are usually modeled by rigid bodies, whereas soft tissues are built by viscoelastic materials [13]. Although these models provide satisfactory insights, they are limited by constitutive data or boundary conditions that represent real anatomy and physiology.

The objective of this study was to develop a sophisticated, MRI-based finite element model to simulate changes in the LAM during vaginal delivery with regard to the cardinal movements of labor.

## Materials and methods

An MRI-based three-dimensional computer model of the bony pelvis, pelvic floor muscles, pelvic floor organs, and fetal head was created. The geometry of these structures was based on live-subject MRI data. The subject was a 25-year-old nulliparous Caucasian woman (BMI 21.9 kg/m^2^), who gave written consent. The reason for choosing nulliparous women for the pelvic model was to reduce the variation from normal anatomy of the LAM subdivisions (especially the iliococcygeus muscle). It cannot be excluded that pregnancy itself is a potential source of anatomical LAM changes, which could be seen after cesarean section. This subject met the following inclusion criteria: no previous vaginal delivery, normal POP-Q points, and absence of PFD symptoms. This study was approved by the local ethics committee from our institution.

The imaging protocol was a high-resolution axial 3 T MRI scan (Phillips Achieva TX series), taken in the supine position. The imaging parameters were as follows: repetition time 5,331 ms, 375 phase encodes, 24-cm field of view, and 2-mm slice thickness, no gap. The pixel dimensions were 0.75 × 1.07 × 2 mm. The pelvic floor model was reconstructed from 130 axial 3 T MRI images. The fetal head model was reconstructed from the head MRI scan of a neonate. There were neurological indications for a MRI brain scan for the 1-day-old neonate (birth weight of 3,600 g) after an uncomplicated vaginal delivery at term. The fetal head model was reconstructed from 104 axial MRI images.

The simulations were focused on the LAM; thus, other organs were neglected. Also, we assumed that the other pelvic organs (bladder, urethra, rectum) were pushed during the delivery without any significant reaction forces.

### Model geometry and finite element mesh

**Fig. 1:**
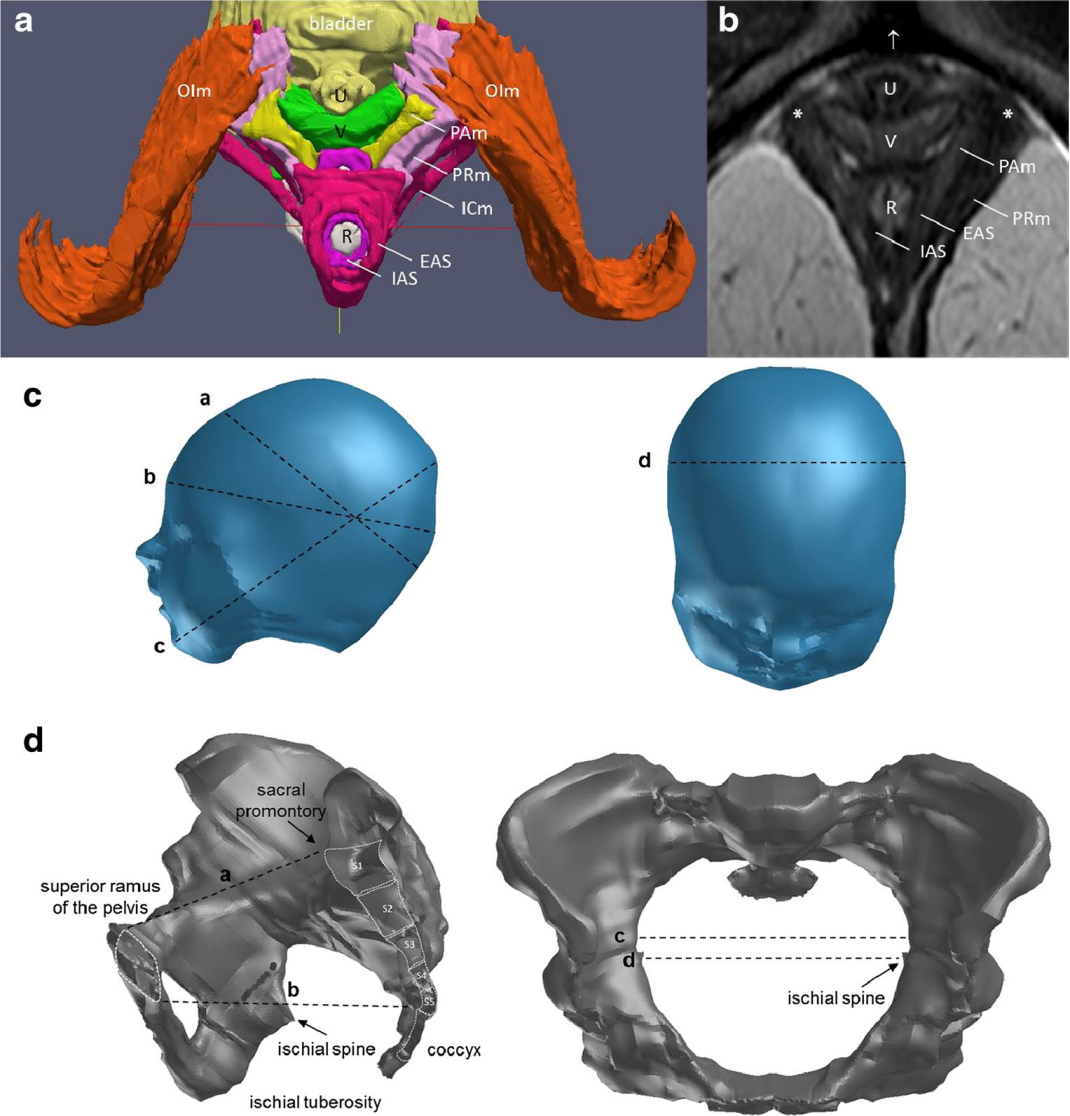
**a** View of the LAM muscle from below. Vulvar structures, perineal membrane, and bony pelvis are not visualized. Internal obturator muscle (*OIm*, *orange*), external anal sphincter (*EAS*, *light red*), internal anal sphincter (*IAS*, *dark purple*), puboanal muscle (*PAm*, *yellow*), puborectal muscle (*PRm*, *light purple*), iliococcygeus muscle (*ICm*, *dark red*), urethra (*U*, *light yellow*), vagina (*V*, *green*), and rectum (*R*, *gray*). **b** Relevant axial MRI shows the urethra (*U*), vagina (*V*), rectum (*R*), and muscles of the levator ani. Note the attachment of the levator ani muscle (*asterisk*) to the pubic bone. The arcuate pubic ligament is shown (*arrow*). **c** The principal dimensions of the fetal head: *a* suboccipitobregmatic dimension = 100.3 mm, *b* occipitofrontal dimension = 105.7 mm, *c* occipitomental dimension = 131.9 mm, *d* biparietal diameter = 93.7 mm. **d** The principal dimensions of the female bony pelvis. Lateral view of the pelvic bones: *a* pelvic inlet = 113.4 mm, and *b* pelvic outlet = 145.1 mm. Axial view demonstrating *c* transverse diameter of the pelvic inlet = 148.8 mm and the *d* interspinous diameter = 146.7 mm

The initial model was reconstructed from MRI using a 3D Slicer (3.0; BWH, Boston, MA, USA). The outlines of the relevant structures were digitized from these consecutive axial MRI scans. The structures included the pelvic bones (anterior and lateral to the innominate bones and posterior to the sacrum and coccyx), internal obturator muscle (OIm), and subdivisions of the LAM (iliococcygeus muscle, arising from the tendinous arch of the levator ani and inserted into the iliococcygeal raphe; puborectal muscle, arising from the pubis and forming a sling behind the rectum; and PVm, arising from the pubis and inserting into the vaginal wall and external anal sphincter [14]), with its connective tissue origin and insertion. The urethra, urinary bladder, vagina, rectum, and internal and external anal sphincters were also outlined in each consecutive axial scan (Fig. 1). The dimensions of the female bony pelvis and fetal head are shown in Fig. 1.

The resulting geometry and mesh were created in HyperMesh (11.0; Altair, MI, USA). Rigid parts, such as the pelvis and fetal head, were constructed with 2D mesh. This mesh included 119,204 triangular elements. Deformable parts, such as muscles, were modeled by 3D mesh, consisting of 179,157 tetrahedral elements. The mean length of all element edges was 2 mm and the global Cartesian coordinate system was used.

### Initial and boundary conditions

The initial (ICs) and boundary conditions (BCs) were considered to simulate the actual behavior of the LAM during vaginal delivery. The BCs represent the structure of the surrounding support of muscle and bone movements, and the ICs reflect the initial position and rotation of all the components observed. The pelvic model was fixed for all degrees of freedom to fit the model in space. The pelvic bones were modeled by rigid bodies, and the material parameters were calculated using a published dataset [15].

Passive connective tissue in the model followed the outlines of relevant structures. The internal obturator muscle (OIm) was fixed to the bony pelvis, also providing support for the LAM. At the beginning of delivery, the LAM remained at rest without any external load. The tendinous arch of LAM (ATML) traverses the anteromedial aspect of the OIm.

To create the LAM model, three pairs of LAMs, based on existing descriptions [14], were included. The PVm and puborectal muscle (PRm) originate from the fibrous enthesis on the dorsal surface of the pubic bone [16, 17]. This section of the muscle is translationally and rotationally fixed. The iliococcygeus muscle (ICm) originates from the ATML. The nodes that represent the parts of the muscle that were attached laterally to the ATML were fixed to the OIm. Thus, their movement depended on elongation of the OIm. The cranial section of the ICm created a horizontal layer that covered the space in the dorsocranial part of the pelvis. Laterally, the muscle is attached to the posterior ilia and dorsal to the sacrum; this part of the muscle is translationally and rotationally fixed.

Nodes along the upper cranial third of the ICm were considered to move only within the plane that was defined by the sacrum and posterior ilia in the inferior and lateral directions. This condition represents LAM support provided by ties, such as ligaments and tendons that were attached to the pelvic walls. All other nodes of the model were unconstrained.

When the MRI-based model of the pelvis and pelvic floor was completed, we used it to estimate stretch ratios in individual parts of the LAM in relation to the descent of the fetal head through the pelvis in the second stage of vaginal delivery. To this end, an entire MRI-based fetal head model was passed incrementally through the pelvic model. The fetal head trajectory was given by the anatomical axis (curve of Carus) and followed the cardinal movements of labor.

**Fig. 2:**
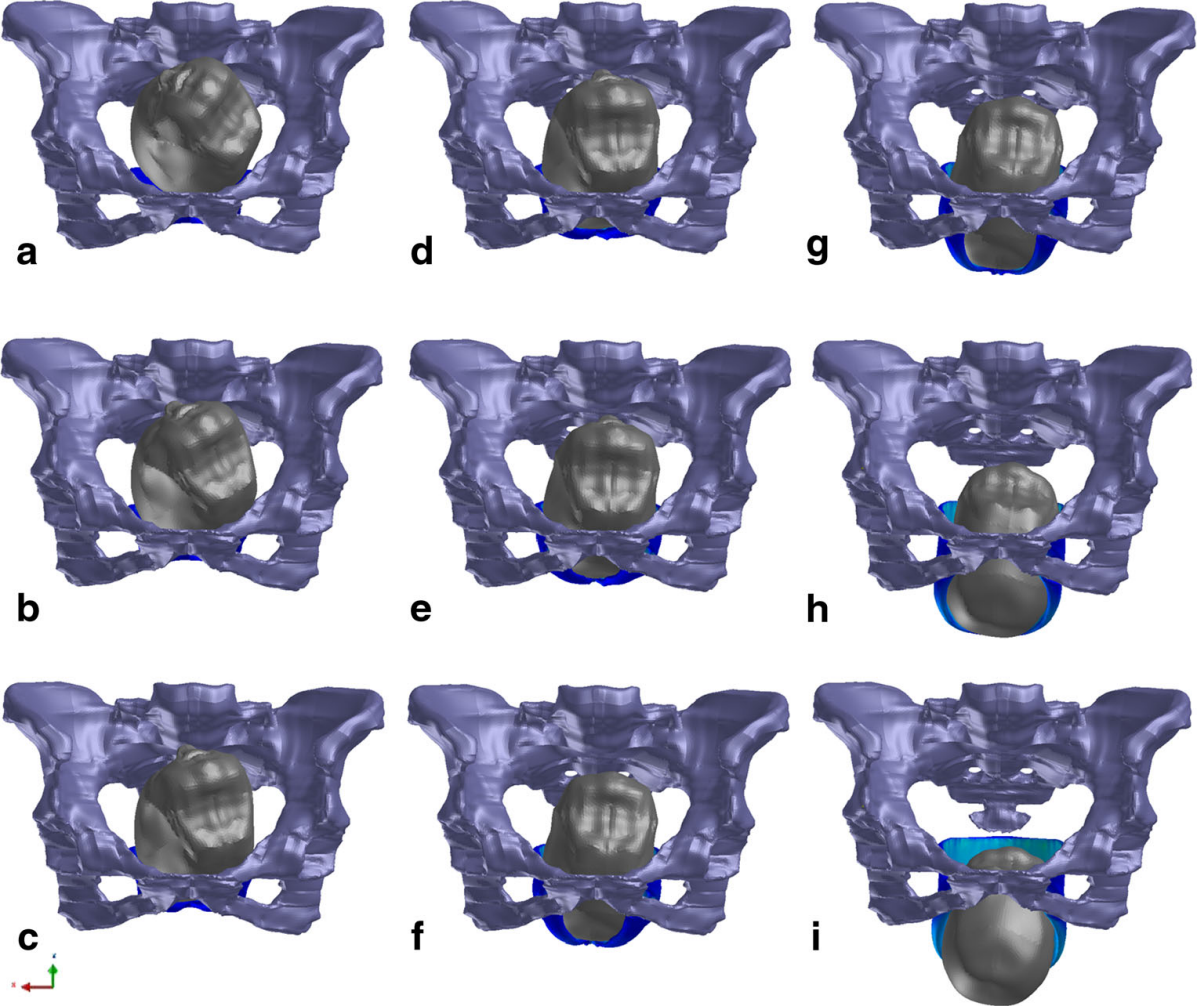
Sequence of nine images showing the simulated effects of fetal head descent and internal rotation on the LAM during the second stage of labor. At the level of the pelvic inlet, the fetal head was in the left occiput anterior position. **a** The head is engaged in the pelvic inlet. The vertex is located 1 cm over the 0 line (station −1). **b** The vertex is located at the level of the ischial spines (station 0). **c**–**g** The sequence of five images shows further fetal head descent and internal rotation 1.0, 2.0, 3.0, 4.0, and 5.0 cm below the line 0 (stations +1, +2, +3, +4, and +5). **h** Beginning of extension. **i**. Complete extension

The fetal head was modeled by rigid bodies without any deformations. At the level of the pelvic inlet, the fetal head was in the left occiput anterior (LOA) position. For station, the pelvis was divided above and below the spines into five levels. Following obstetric convention, the internal rotation moved the occiput from its original position anteriorly toward the symphysis pubis. At the level of the ischial spines, the internal rotation was completed when the vertex was located below the midpelvic plane (station + 3; Fig. 2).

**Fig. 3:**
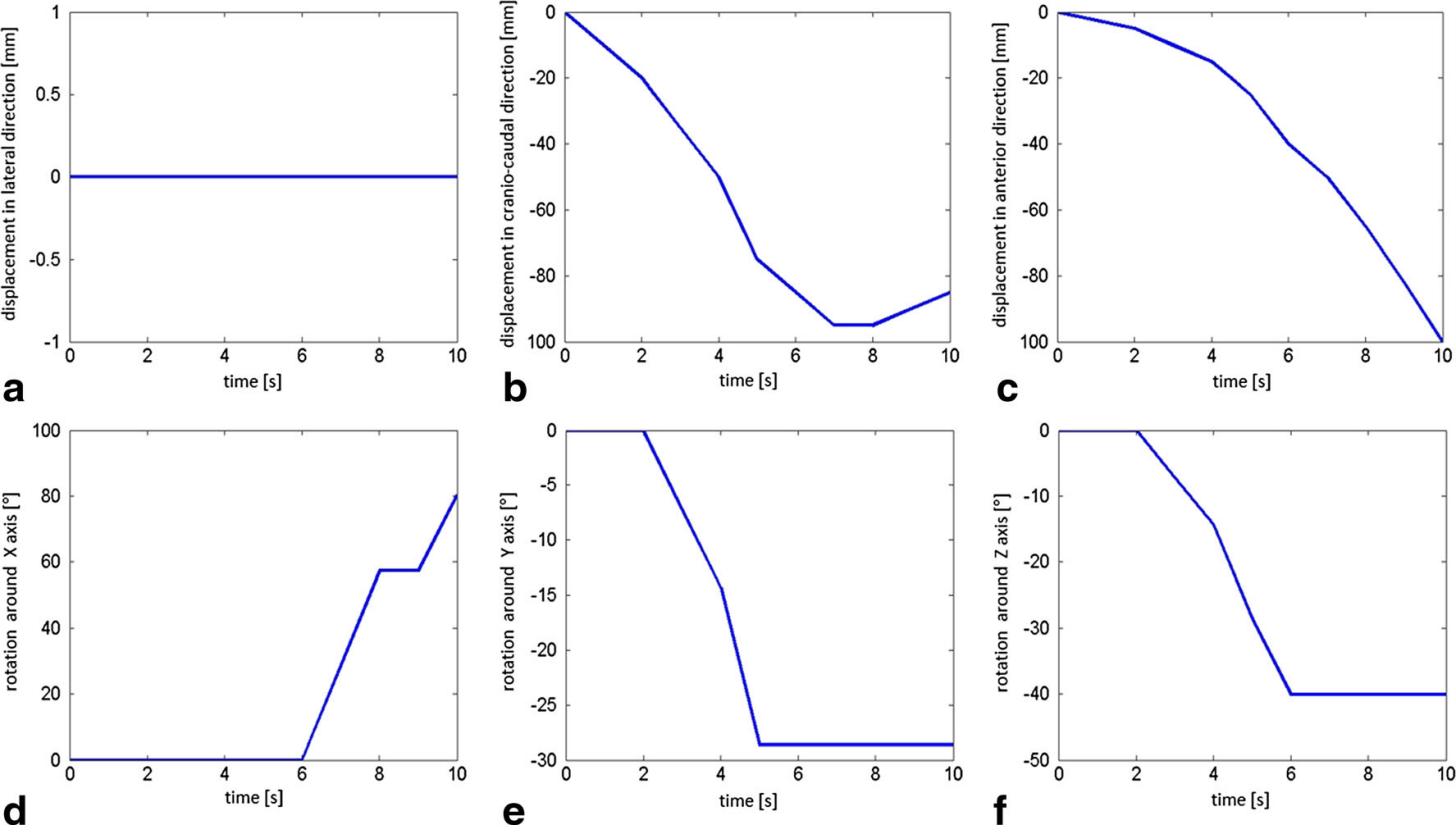
Biomechanical description of fetal head movements (displacement, rotation, extension) in the birth canal. **a** During the internal rotation, there was no lateral shift to the left or right side. **b** In the craniocaudal direction, the trajectory of the fetal head model reflected the curve of Carus. **c** The displacement in the anterior direction from the beginning to the end of the simulation was 100.0 mm. **d** After the internal rotation, the sharply flexed head underwent extension of 80.25°. **e** The head initially deviated 28.66° to the right hand side; thus, it was straightened. **f** The vertex of the fetal head model entered the pelvis in the left occiput anterior position. The internal rotation was completed at station +3. The occiput rotated around the z axis from the left anterior position 40.13 degrees anteriorly

A biomechanical description of the fetal head movements in the birth canal is shown in Fig. 3.

### Material properties

During vaginal delivery, the LAM undergoes extremely large deformations [18]. Thus, viscoelastic nonlinear Ogden material model was used [19, 20].

During muscle tension, the homogeneous deformations were considered: *x*_*1*_ = *λ*_*1*_ *X*_*1*_, *x*_*2*_ = *λ*_*2*_ *X*_*2*,_ *x*_*3*_ = *λ*_*3*_ *X*_*3*_, where *X*_*1*_, *X*_*2*_, *X*_*3*_ are coordinates identifying material particles in an unstressed configuration; *x*_*1*_, *x*_*2*_, *x*_*3*_ are the corresponding coordinates after the deformations; and the coefficients λ_1_, λ_2_, λ_3_ are constants that are referred to as the principal stretches of deformation. The deformation gradient, ***F***, in terms of principal stretches, is then:

$$ \boldsymbol{F}=\left[\begin{array}{ccc}\hfill {\lambda}_1\hfill & \hfill 0\hfill & \hfill 0\hfill \\ {}\hfill 0\hfill & \hfill {\lambda}_2\hfill & \hfill 0\hfill \\ {}\hfill 0\hfill & \hfill 0\hfill & \hfill {\lambda}_3\hfill \end{array}\right]. $$

According to the theory of viscoelasticity, the material properties are described by a strain energy function, *W*, depending symmetrically on *λ*_*1*_, *λ*_*2*_, and *λ*_*3*_. For an incompressible material, the principal stretches satisfy the constant *λ*_*1*_ *λ*_*2*_ *λ*_*3*_ = 1. For Ogden material, the strain function is given by the following formula [21]:

$$ \begin{array}{ccc}\hfill W={\displaystyle \sum_{i=1}^3{\displaystyle \sum_{j=1}^N2\frac{\mu_j}{\alpha_j}\left({\overline{\lambda}}_i^{\alpha_j}-1\right)+\frac{K}{2}{\left(J-1\right)}^2}},\hfill & \hfill {\overline{\lambda}}_i={J}^{-\frac{1}{3\lambda }},\hfill & \hfill J= \det \boldsymbol{F},\hfill \end{array} $$

where *μ*_*j*_ [*MPa*] and *α*_*j*_ [-] are the Ogden parameters and *N* is the number of terms in the Ogden series. For our purposes, *N* = *2*. Assuming uniaxial tension, the unknown variables are λ_2_ = λ_3_ = λ_1_^−1/2^ and J = 1, and thus, the parameter *K* is not significant. For the parameter estimation, data from the uniaxial tensile test of the LAM from a female cadaver were used [15]. Stress–strain curves were used to identify parameters in the specific strain energy function by the least squares method. For the case of uniaxial tension, the nominal stress–stretch relation for the Ogden material can be written as:

$$ P=\frac{\delta W}{\delta \lambda }={\displaystyle \sum_{i=1}^N2{\mu}_i\left({\lambda}^{\alpha_i-1}-{\lambda}^{-\frac{1}{2}{\alpha}_i-1}\right).} $$

*P* is nonlinear, and thus, the nonlinear least square method is used to determine the Ogden parameters. The method consists of minimizing the error in stress:

$$ E=\frac{1}{2}{\displaystyle \sum_{i=1}^{ND}{\left({p}_i^{test}-{p}_i^{derived}\right)}^2,} $$

where *P*_*i*_^*test*^ is the measured nominal stress for the nominal strain measure, *P*_*i*_^*derived*^ is the stress obtained from the strain energy function, and *ND* is the number of data points. For our purposes, the *lsqcurvefit* function in the Optimization Toolbox of MATLAB (The MathWorks, Natick, MA, USA) was used. The lower and upper bounds were added to ensure the inequality considered for all parameters:

$$ {\displaystyle \sum_{i=1}^N{\mu}_i{\alpha}_i>0.} $$

This condition ensures stable behavior of small deformations from the initial configuration.

The material properties of the LAM were fitted using the least squared method and published stress–strain characteristics [15]. The LAM elastic parameters μ and α were: μ1 = 8 · 10^−5^ MPa, μ2 = 1.7 · 10^−4^ MPa, α1 = 1.81, and α2 = 17.25.

The density of general mammalian skeletal muscle tissue is approximately 1.06 kg/l [21]; the value of Poisson’s ratio was 0.499, as usual. The friction between the fetal head and female muscles was neglected, assuming that the contact is sufficiently lubricated during the actual delivery.

### Stress distribution in the LA

Vaginal delivery, scaled in seconds, was simulated with an optimal initial position and internal rotation of the fetal head. The distribution of von Mises stress in the LAM was analyzed by the finite element method using Virtual Performance Solution (VPS 9.0; ESI Group, Paris, France).

## Results

The relationship between fetal head descent and the simulated von Mises stress distribution that was generated in selected LAMs is shown in Table 1. The descending fetal head initially distends the ICm portion of the LAM at station −3. The mean stress value at this station was 0.13 MPa. At station −3, the other portion of the LAM did not experience changes in stress. The greatest increase in stress in this area of the LAM occurred during fetal head extension; the mean values were 4.91–7.93 MPa.

**Table 1:** The relationship between fetal head descent and simulated distribution of von Mises stress generated in selected levator ani muscles. Our fetal head was simulated with a biparietal diameter of 93.7 mm, representing the 50th percentile

	BPD diameter 93.7 mm											
	Von Mises stress distribution (MPa)											
	Upper dorsal LAM portion (iliococcygeus muscle)				Left attachment anteromedial LAM portion (pubovisceral and puborectal muscle)				Distal posteromedial LAM portion (puborectal muscle)			
Head descent (cm)	Mean	Minimum	Maximum	±SD	Mean	Minimum	Maximum	±SD	Mean	Minutes	Maximum	±SD
–3	0.13	0.00	0.25	0.13								
–2	0.15	0.00	0.30	0.15								
–1	0.01	0.00	0.02	0.15	0.35	0.00	0.70	0.35				
0	0.22	0.00	0.44	0.22	0.37	0.00	0.73	0.36				
+1	0.25	0.19	0.31	0.06	0.39	0.36	0.42	0.03	0.02	0.00	0.04	0.02
+2	0.98	0.19	0.31	0.46	0.41	0.34	0.45	0.06	0.03	0.00	0.07	0.04
+3	1.59	0.91	2.26	0.67	0.25	0.07	0.43	0.18	0.05	0.00	0.11	0.05
+4	2.22	0.63	3.82	1.59	0.18	0.11	0.26	0.07	0.08	0.00	0.16	0.05
+5	3.02	1.11	4.93	1.91	0.71	0.22	1.20	0.49	0.71	0.05	1.38	0.66
+6	3.88	0.36	7.77	2.63	3.38	1.13	5.36	1.54	3.79	1.93	7.16	1.94
+8	4.91	0.00	10.00	4.11	4.14	0.08	8.66	2.98	10.19	7.22	13.24	2.19
+10	6.56	0.02	0.02	13.60	11.38	4.10	17.04	4.64	12.39	0.43	23.41	9.00
+11	7.78	0.28	16.13	6.59	27.46	16.31	35.75	7.80	12.92	0.85	26.33	10.59
+12	7.93	0.79	5.17	5.16	18.01	4.00	28.08	8.98	16.89	0.23	35.00	14.07

The largest absolute values of stress on the LAM were induced in sections of the muscle arising from the pubic bone—the PVm and PRm portions of the LAM. The mean value in this area was 27.46 MPa at the outset of fetal head extension.

**Fig. 4:**
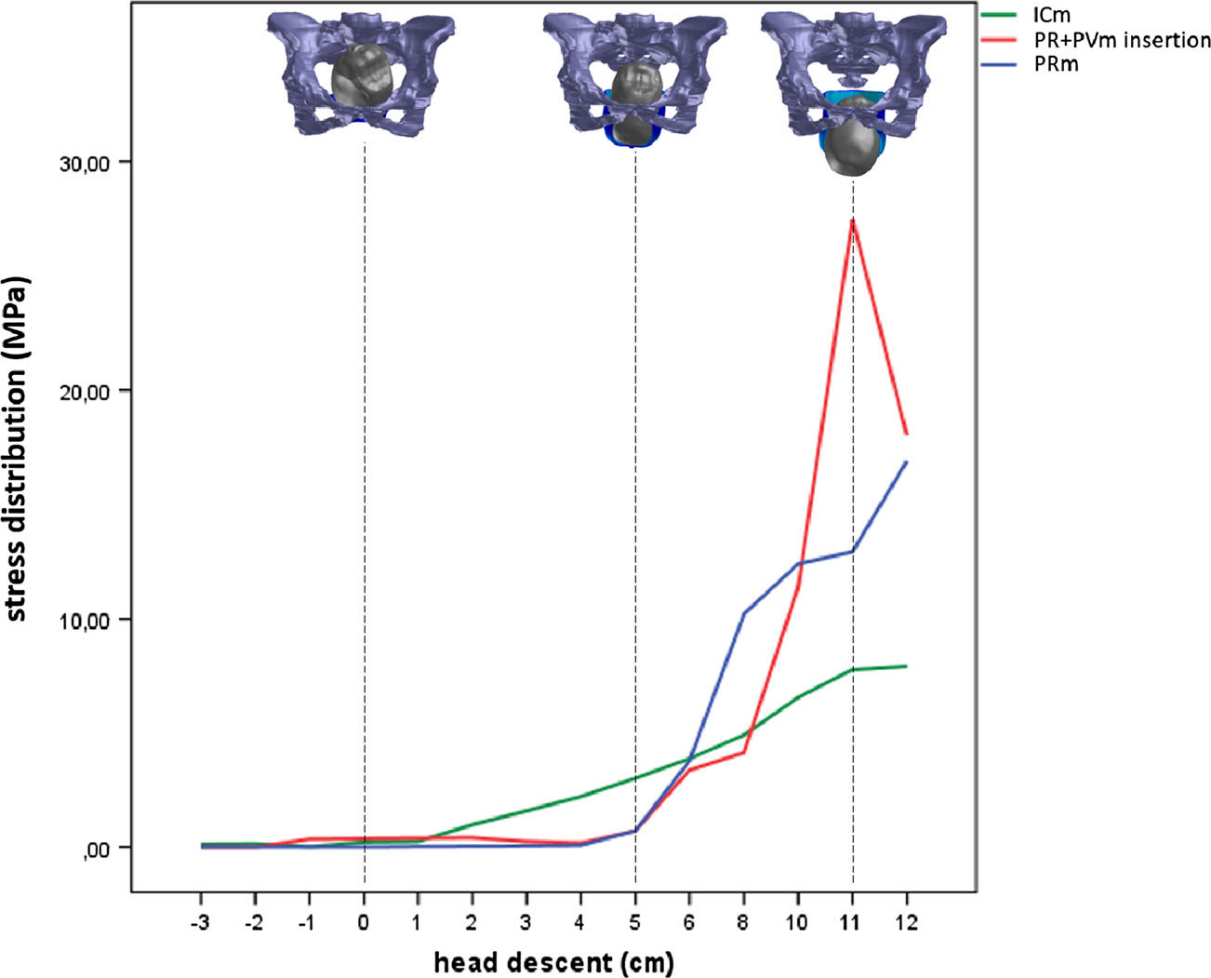
The relationship between the distribution of von Mises stress and head descent in selected LAM subdivisions. The labels in the right hand corner identify the ICm, portions of the muscle that arise from the left pubic bone, the PVm and PRm areas of the LAM (PR + PVm insertion), and the posteromedial section of the PRm. The greatest stress induced by the engaged head was localized to the LAM portion that arises from the pubic bone: PRm and PVm

The posteromedial portion of the PRm began to undergo changes in stress at fetal head station +1. The mean stress value was 0.02 MPa, which was the lowest value compared with other areas of the LAM at this station. This was the last LAM subdivision to undergo changes in stress. The comparison of von Mises stress and head descent by LAM subdivision is shown in Fig. 4.

**Fig. 5:**
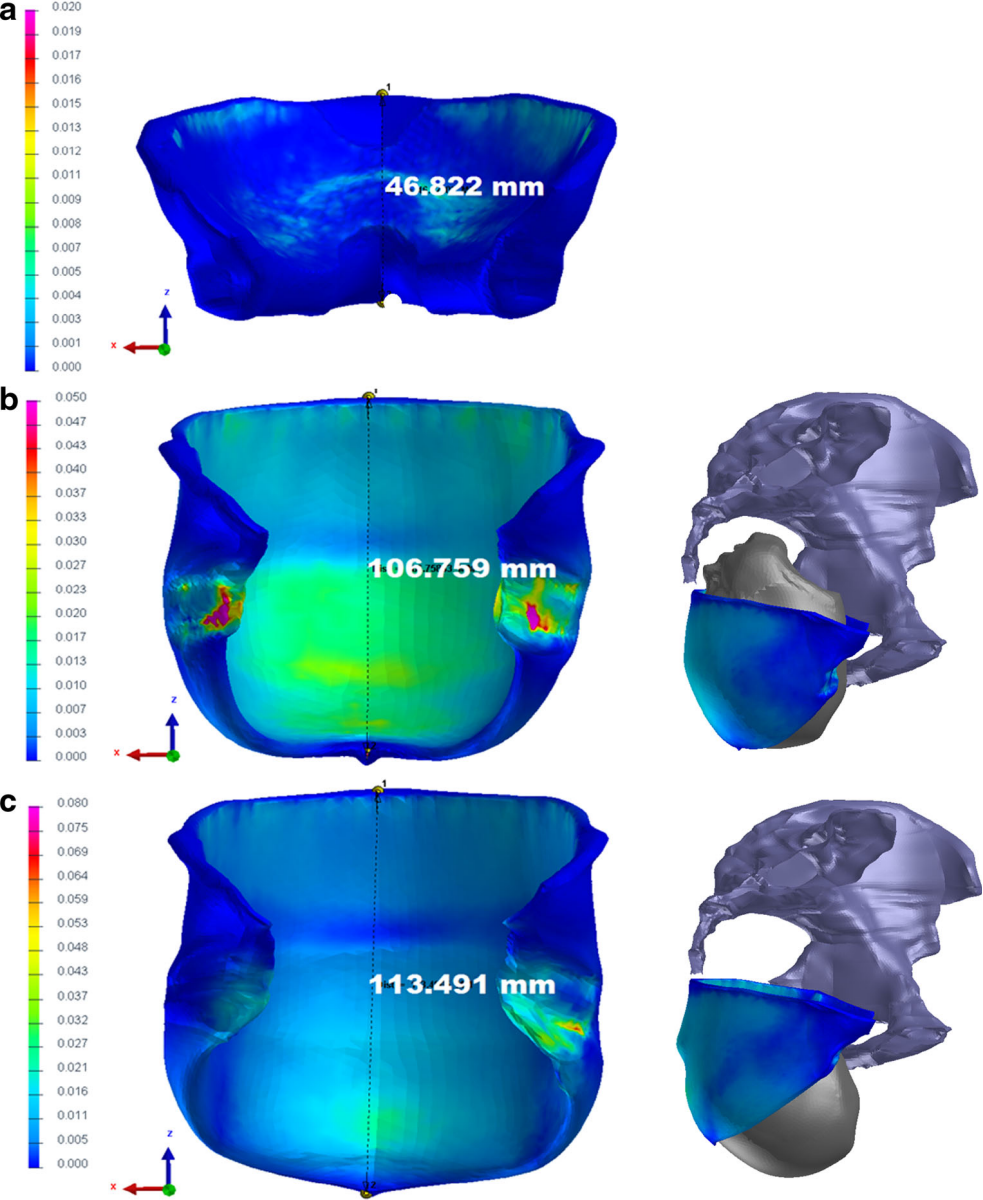
Color-coded view of the levator areas demonstrating the distribution of von Mises stress at various levels of fetal head model descent. All stress values are given in GPa. Deformation in the caudal direction, expressed in millimeters. The frontal view (*left*), shows the stress distribution in the whole LAM. The side view (*right*) represents the actual position of fetal head with regard to the pelvis. The figure itself is only visual support for the written description. **a** The head is engaged in the pelvic inlet, and the vertex lies 1 cm below the 0 line (station +1). At this level, the initial stress changes are presented in the upper third and the central part of the ICm and predict muscle elongation in the caudal direction. **b** The vertex is placed below the +5 line. The fetal occiput is in contact with the inferior margin of the symphysis pubis, and head extension starts. Parallel to the caudal elongation of the ICm, the von Mises stress increases in the central part of the ICm (*green area*). The greatest stretch is induced in the PVm with the PRm originating from the dorsal surface of the pubic bone (marked with *pink* and *red*). **c** In the last position, extension is complete. The distribution of stress in the PVm with the PRm originating from the dorsal surface decreases. The most distal point on the PRm (*asterisk*) loop is displaced by a maximum of 66.66 mm in the caudal direction as a result of distension by the fetal head

The color-coded versions of the distribution of simulated von Mises stress in the LAM are shown for three fetal head positions along the anatomical pelvic axis in Fig. 5. The most distal point on the PRm loop was displaced by a maximum of 66.66 mm in the caudal direction as a result of distension by the fetal head. The LAM was elongated in the mediosagittal plane by nearly 2.5 times versus its initial resting position.

## Discussion

Our study confirmed the assumption of extremely large deformations in the LAM during the second stage of vaginal delivery. The location of deformation was nonhomogeneous, and the values depended on fetal head placement.

Unlike other studies, in which the fetal head model has been oversimplified as a sphere [4, 22], we used the geometry of an actual head from MRI. The effect of fetal head molding, which normally assumes a 20 % reduction in diameter [23], was not calculated in the simulation. Lien et al. considered the effect of molding, but the fetal head was simulated with three different spheres [4]. The movements of the fetal head during birth were simulated during the cardinal movements of labor. In other studies that used a sphere, the cardinal movements of labor could not be reflected.

Based on anatomical descriptions, three pairs of LAMs (PRm, PVm, ICm) were included in constructing our 3D MRI-based pelvic floor model [14]. We also had precise knowledge of the anatomy of the origin of the PVm and specific vaginal birth-induced injuries that typically occur on the inner surface of the pubic bone [16]. There is also growing evidence on the function of the LAM in the activity of the pelvic floor, indicating that the PVm and PRm participate in hiatal closure. The muscle fibers of the ICm have a similar direction to those of the PVm; thus, they should have additional lifting effects [24]. According to these findings, the effects of birth-related injury to the LAM on its function depend on the region that experienced trauma. Our model demonstrates that the descending fetal head initially distends the ICm portion of the LAM at station −3. Concurrently, the PVm with the PRm did not demonstrate a distribution of von Mises stress. The maximum stretch to the posteromedial portion of the ICm was observed when the vertex was placed below the +5 line, when the fetal occiput was in contact with the inferior margin of the symphysis pubis and head extension began. The maximal stress values in this area were lower than those in the PVm and PRm. This deformation indicates that injury to the LAM also involves the ICm, but such defects are seen less commonly (2 %) in primiparous women compared with pubovisceral defects [25]. One explanation for the smaller extent of stretch in the ICm is the difference in length and insertion origin of the muscle fibers [4]. During fetal head descent, the ICm fibers separate from one another in the direction of their longitudinal fibers, resulting in biaxial stretching of the muscle bands. During this stretch downward, certain muscle fibers could be avulsed from their origin, whereas others may be denervated, but the overall connections of the muscle to the bone may remain intact.

During the simulation, we used only 1 degree of head flexion, the smallest head diameter was present continuously at the birth canal. The decrease in fetal head flexion could be associated with higher opposing forces in the ICm against fetal head descent [26], which can contribute to malfunction. The maximal mean stress in the ICm was 7.93 MPa, which corresponds to published results [27].

The highest stretch values were induced in the part of the LAM that arises from the pubic bone, correlating with previous simulation by Lien et al. [4], who concluded that the medial-most section of the pubococcygeus muscle is at greater risk of birth-related injury during the second stage of labor than any other LAM subdivision. The short initial length of the muscle component and the location of the muscle origin near the midline could be factors of birth-related injury. In this paper individual LAM muscle bands have been identified and the stretch ratios in each LAM subdivision band were also analyzed. Our model was unable to differentiate the specific portion of the LAM that arose from the affected pubis. However, according to observations that the points of attachment of muscle fibers often overlap and are not entirely parallel, and because the resulting movement of structures depends on the interaction between muscles, we suggest that this is not a severe limitation.

Following a woman’s first vaginal birth, muscle defects are seen in 18 % of primiparous women. The appearance of these muscle defects in the ventral component of the pubic portion of the muscle correlates with significantly smaller cross-sectional areas compared with women with intact muscles [28]. The presence of abnormalities in this part of the LAM in the postpartum years supports the hypothesis of a stretch-related injury mechanism, although we cannot exclude the possibility of other injury modes. The highest stress in this area in our model was 35.75 MPa, which was nearly 10 times higher than in a study by Jing et al. [27]. The maximum values of stress of the PVm complex in our model were obtained during fetal head model extension. In Lien et al., the highest stretch ratio was measured in the pubococcygeus muscle with 9.9 cm of head descent, although the fetal head was simulated with a sphere. The actual fetal head geometry that was used in our setting represents a more realistic simulation.

At the same time as the portion of the LAM arose from the pubic bone, the stress values began to increase significantly in the posteromedial sections of the PRm when the vertex was placed at station +5. The maximum mean stress in the posteromedial inner area of the PRm was 16.89 MPa when the extension was completed, correlating with previous simulation, in which the maximum stretch was localized specifically to the identical area of the PRm [12].

In our study, the largest LAM stretch ratio was 2.5 in the inferior direction, consistent with the value of 2.85, as suggested by the model [12], and within the range of 1.62–3.76, based on a statistical study of 227 women [29].

Some virtual models of the female pelvic floor still use oversimplified material. In contrast, we used the most suitable Ogden model. Another advantage of our model is that the OIm was involved, contributing significantly to the correct specification of the BCs. Another novel finding was the comprehensive description of the distribution of von Mises stress. Most studies have modeled the lower part of the LAM, merely presuming that the highest stresses are generated solely in this area. Our study considered the entire LAM structure to describe the general stress that was experienced. Another advantage is that the cardinal movements of labor of the real fetal head were simulated.

A limitation of this model is that the fetal head and female pelvis were modeled by rigid bodies without any deformations. The second limitation is that the head was driven at a given trajectory with homogeneous progression. Linear progress is used as a simplification of real labor in our numerical simulation. We plan to consider more realistic motion of the fetal head (progress and standstill) in our future studies. The third limitation is that the LAM was modeled by a continuous structure to simplify the task and reduce the computation time. To increase the accuracy of the model, this muscle should be separated into substructures, especially LAM subdivisions that arise from the pubic bone. Furthermore, with regard to the behavior of the material, the Ogden parameters were computed using a dataset from a uniaxial test of cadaver tissue. However, using cadaveric samples creates several problems (postmortem changes, advanced age of the donors, postnatal changes).

It is important to note that the present modeling technique is based on a single adult test person and a single neonate. Further research studying age categories and racial groups in a larger cohort seems justified. Finally, active contraction of the LAM was neglected and the MRI scan was conducted in a supine position. This position with adducted legs does not always reflect the normal course of labor. Despite its limitations the present model eliminates some of the deficiencies of existing models. In the future, this model will be used to calculate stress distribution in the LAM depending on fetal head size, pelvic dimensions, and obstetric complications.
